# Political polarization threatens fairness and reciprocity in the USA

**DOI:** 10.1038/s41598-026-42697-4

**Published:** 2026-03-28

**Authors:** Detlef Fetchenhauer, Thomas Graczyk, Stephan Joel Triemer, Anne-Sophie Lang, Sebastian Winterhagen, Simon Kemp

**Affiliations:** 1 Department of Sociology and Social Psychology (DSS), Faculty of Management, Economics and Social Sciences, University of Cologne, Cologne, Germany; 2 School of Psychology, Speech and Hearing, University of Canterbury, Christchurch, New Zealand

**Keywords:** Polarization, Trust, Reciprocity, USA, Politics and international relations, Psychology, Psychology

## Abstract

An increase in political polarization in the USA has been reported by many researchers using different kinds of data (e.g., attitudinal, affective and behavioral measures). Here, we report the results of three incentivized experimental studies (with a total of 1842 participants) in which participants had to choose how to divide money given to them by a political opponent (decisions were made for actual money of up to $11). In Study 1, participants could choose whether to act trustworthily (i.e., to share the money evenly or keep it for themselves). In Study 2, participants had no financial incentives not to reciprocate the trust placed in them (i.e., they did not earn their own benefits from harming their interaction partner). In both Study 1 and Study 2, more participants actively harmed a political opponent than a political co-partisan or a person with an unknown political affiliation. Most strikingly, such discrimination was not only governed by disliking members of the other side, but also perceived as justified moral aggression (i.e., it was regarded as the behavior that *should* be chosen). In Study 3, a previously well-established intervention to mitigate affective political polarization increased the likability of opponents but did not reduce discriminatory reciprocity. In all three studies, when compared with an anonymous interaction partner participants only slightly favored political affiliates but strongly discriminated against political opponents. In this, our results were highly symmetrical: Democrats and Republicans did not systematically differ in their willingness to act fairly towards each other.

## Introduction

“Human beings are consistent in their codes of honor, but endlessly fickle with reference to whom the codes apply.” (E.O. Wilson)^1^.

The 2024 presidential election in the United States was harsh and hostile on both sides. Donald Trump called his opponent Kamala Harris a “lunatic”^2^ (amongst many other things); both candidates accused each other of being a “threat to democracy”^3,4^ and “fascist”^5,6^.

It was not only the presidential candidates who disliked each other. The same seemed to be true for their Democratic and Republican voters. For many decades now it has been observed that political polarization in the USA is increasing^7–10^. Attitudes towards major political issues are getting more and more dissimilar^11^, and political opponents experience each other increasingly as “immoral”, “dishonest,” and “unintelligent”^12^. Furthermore, behavioral scientists have shown that participants cooperate less with political opponents than with co-partisans across various economic games. This includes studies using Prisoner’s Dilemmas^13,14^, Dictator Games^15–17^, Public Good Games^18^, the Equality Equivalency Test^19^, and the Trust Game^17,20^. However, previous studies did not reveal the specific reasons for these results. Do voters want to favor political co-partisans or do they want to discriminate against political opponents? Do they refuse to cooperate with political opponents out of pure self-interest or out of emotional disdain?

Here, we investigated the behavior of Democrats and Republicans in dynamic interactions with actual money, in which they were given the choice to reciprocate trust given to them by (a) a political affiliate; (b) a political opponent; and (c) an anonymous interaction partner they had no information about. Our studies were conducted close to the 2024 presidential election (in the Autumn of 2024), and thus provide insight into voters’ feelings, attitudes, and behavior in the midst of the election.

In Study 1, participants had to make decisions in a binary trust game^21–23^ involving two interaction partners (Person A and B). Participants had to make decisions with regard to both positions. Person A (the trustor) was given $2.50, which they could either keep for themselves or send to Person B. If Person A kept the money, Person B did not receive any money at all. If Person A sent the money to Person B, Person B (the trustee) had two options. Either Person B could decide that both they themselves and Person A would receive $3.75, or Person B could decide to receive $11 and for Person A to get nothing. (Note that participants were compensated in British pounds (GBP) through the Prolific platform (https://www.prolific.com/). To enhance clarity for an international audience, monetary values are reported in U.S. dollars (USD) throughout the manuscript.)

The trust game captures the essence of any trust situation: Is Person A willing to make themselves vulnerable to the goodwill of Person B? Is Person B willing to honor the trust given to them by Person A^24,25^? Previous studies have shown that under conditions of total anonymity, a majority of Persons A sends their money to Person B, and a majority of Persons B act in a trustworthy manner (i.e., they share the money evenly between themselves and Person A). Indeed, a majority of participants indicate that in the position of Person A, they *should* trust Person B and that in the position of Person B, they *should* reciprocate trust given to them by Person A^22,24,26^. Thus, people feel obliged to be both trustful and trustworthy – even in one-shot interactions under conditions of total anonymity.

The trust game has been used before to demonstrate the consequences of political polarization^17,20,27–31^. Results have consistently shown that political partisanship in the United States influenced the behavior of Person A (trust), but not Person B (trustworthiness)^20,31^. However, most previous studies contained methodological drawbacks: (1) In some studies, only the behavior of Person A (trust) was reported, but not the behavior of Person B (trustworthiness)^17,28,29^; (2) many studies only measured hypothetical decisions and no actual money was involved^17,27^. Those studies that did adequately report the behavior of Person B^20,31^ did not find discrimination of political opponents. Study 1 aimed to clarify whether this still is the case.

However, in the trust game it is not possible to determine the motivational basis for the behavior of both Person A and Person B. As Person A, not to trust might be driven by an unwillingness to interact with a political opponent *or* by the fear of being exploited. Not being trustworthy towards a political opponent as Person B might be driven by the desire to actively harm Person A or by pure self-interest.

Going beyond the trust game, in Study 2, to adjust for this ambiguity, we employed a new paradigm that we call the *nastiness game*. As in the trust game, Person A was given $2.50 that they could either keep for themselves or send to Person B. If Person A kept the money, Person B received none (as in Study 1). If Person A sent the money to Person B, Person B could decide whether Person A was to receive $3.75 or nothing at all. Whatever Person B decided, Person B always received $2.50. The major difference between this newly developed game and the standard trust game is the potential motivation for Person B not to reciprocate Person A’s trust. When Person B keeps all the money for themselves in a trust game, this might be driven by simple self-interest. However, Person B does not gain materially from being uncooperative in our newly developed paradigm, and their only motive not to reciprocate is to purposefully harm Person A and thus discriminate against a political opponent. Therefore, we coined our newly developed paradigm the *nastiness game*. In a study unrelated to this research, in a nastiness game played under conditions of total anonymity, participants were more trustful as Person A and reciprocated trust more often as Person B than they did in a trust game (unpublished data; see OSF).

Extending Study 2, in Study 3, we tested whether a simple and cost-effective intervention that in a previous study successfully reduced partisan animosity would also be able to increase reciprocity in our newly developed paradigm (i.e., the nastiness game).

In all three studies, we let participants interact with different interaction partners: (1) a person voting for the same political party; (2) a person voting for the opposite political party; and (3) a person whose political preferences were unknown to them. In all three studies, all participants had full transparency and mutual knowledge of their interaction partner’s political affiliation. This enables us to operationalize discrimination in a straightforward manner: A participant was classified as discriminating when they reciprocated trust to a co-partisan, but not to a political opponent.

Going beyond these behavioral data, we also measured participants’ motivation to discriminate against a political opponent. There is a large body of research on potential reasons for ingroup-bias^32–34^. Here, we focused on participants’ hedonic preferences (*want*) and especially on participants’ normative beliefs and perceived moral obligations (*should*): “How do you think you should decide as Person B?” (a value of 1 indicated that one should definitely not send money to Person A and a value of 7 indicated that one should definitely send money to Person A). Did people discriminate against their political opponents with a bad conscience or did they think that discrimination was the morally right thing to do?

In all three studies, decisions were not hypothetical; every tenth participant made one of their decisions for actual money. Research on social preferences in economic games consistently shows that participants’ behavior does not differ substantially between a randomized payment method (e.g., paying every tenth participant) and a non-randomized payment method (e.g., paying all participants)^35–37^.

## Results

### Study 1

**Fig. 1:**
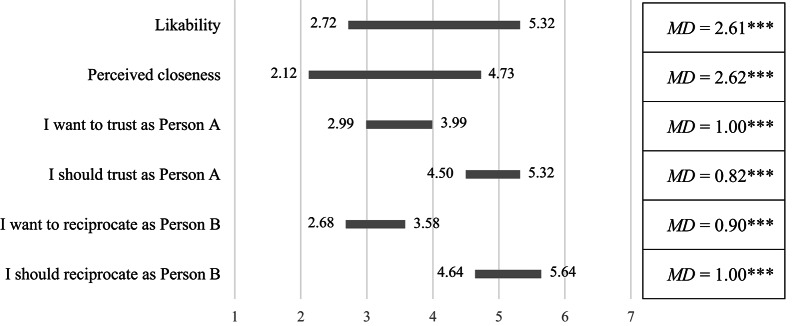
Rounded means (and mean differences) of participants’ perceptions of likability and closeness, as well as what participants wanted and felt they should do as Person A and B toward the political out-group and in-group. Higher values indicate greater likability, closeness, stronger *wanting* to trust (1 = *I want to keep the $2.50* to 7 = *I want to send the $2.50 to Person B*) and feeling one *should* trust (1 = *I should keep the $2.50* to 7 = *I should send the $2.50 to Person B*) as Person A and stronger *wanting* to reciprocate trust (1 = *I want to keep the $11* to 7 = *I want to keep $3.75 and send $3.75 back to Person A*) and feeling one *should* reciprocate trust (1 = *I should keep the $11 and send $0 to Person A* to 7 = *I should keep $3.75 and send $3.75 back to Person A*) as Person B. All values regarding the out-group are on the left side of the bar; all values regarding the in-group are on the right side of the bar. Asterisks indicate the significance of mean differences (in-group–out-group), as calculated using paired samples *t*-tests at the two-sided *p* < 0.05 level (*), at the *p* < 0.01 level (**), and at the *p* < 0.001 level (***).

Participants reported perceiving themselves as significantly closer to an interaction partner sharing their political affiliation than to one of an opposing party (*M* = 4.73, *SD* = 1.49 versus *M* = 2.12, *SD* = 1.20, *t*(345) = 26.98, *p* < 0.001, *d* = 1.45, 95% CI[1.29, 1.63]). Likewise, participants reported liking the in-group significantly more than the out-group (*M* = 5.32, *SD* = 1.10 versus *M* = 2.72, *SD* = 1.30, *t*(345) = 27.20, *p* < 0.001, *d* = 1.46, 95% CI[1.33, 1.62]). Thus, participants indicated closeness and affection towards a political affiliate, and they indicated distance and distaste towards a political opponent (see Fig. 1).

**Fig. 2:**
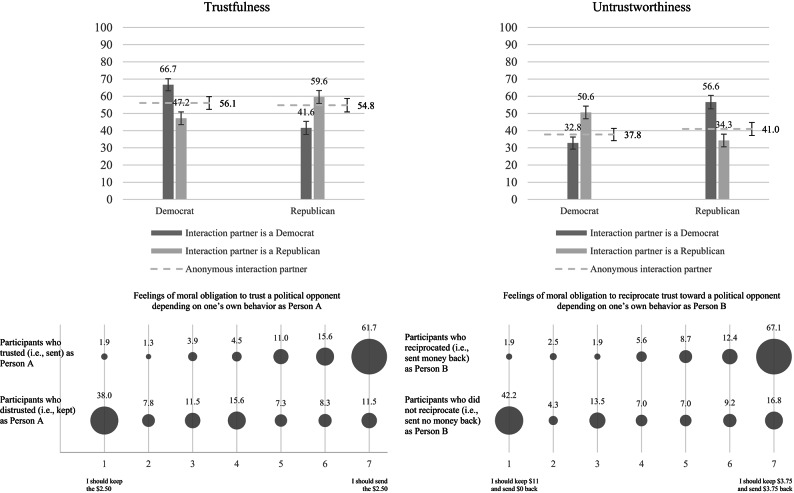
Proportion of participants deciding to send the money as Person A (upper, left) or keep the total amount as Person B (upper, right) by own party and the interaction partner’s party. The lower part of the Figure highlights feelings of moral obligation towards a political opponent as regards trust (lower, left) and reciprocity (lower, right). Error bars depict standard errors.

Across both party affiliations, as Person A, participants sent the money more often to a political affiliate (63.3%) than a political opponent (44.5%) (χ^2^(1) = 53.20, *p* < 0.001) (see Fig. 2). For both Democrats and Republicans, this was mainly driven by out-group discrimination: Each trusted someone of undisclosed party affiliation significantly more often than a political opponent (*OR* = 3.57, *p* = 0.003, 95% CI[1.30, 9.78] and *OR* = 5.80, *p* < 0.001, 95% CI[2.04, 16.52], respectively). Beyond that, Democrats also trusted an anonymous interaction partner less than a political affiliate (*OR* = 0.20, *p* = 0.001, 95% CI[0.07, 0.59]), whereas Republicans showed no comparable in-group favoritism (*OR* = 0.53, *p* = 0.117, 95% CI[0.21, 1.39]). In line with that, further analyses showed that participants expected more political affiliates to be trustworthy than political opponents (*M* = 49.04%, *SD* = 25.28% versus *M* = 24.60%, *SD* = 21.93%, *t*(345) = 16.87, *p* < 0.001, *d* = 0.91, 95% CI[0.80, 1.03]).

*However, were participants right in their assumptions that their political opponents would act less trustworthily towards them?* Yes, they were (see Fig. 2). Overall, in the position of Person B, only 33.5% did not share the money with a political affiliate; however, a majority of 53.5% did not share it when paired with a political opponent (χ^2^(1) = 58.53, *p* < 0.001). Both Democrats and Republicans displayed out-group discrimination in that they significantly less often kept all the money for themselves with someone of undisclosed affiliation than with a political opponent (*OR* = 0.10, *p* < 0.001, 95% CI[0.03, 0.36] and *OR* = 0.09, *p* < 0.001, 95% CI[0.03, 0.30], respectively). Neither Democrats nor Republicans displayed in-group favoritism: They did not keep the money significantly more often with an anonymous interaction partner than with a political affiliate (*OR* = 2.56, *p* = 0.140, 95% CI[0.82, 7.92] and *OR* = 2.95, *p* = 0.057, 95% CI [0.98, 8.92], respectively). All mixed-effects binary logistic regression analyses were conducted in R using the lme4 package^38^, with fully specified models using Adaptive Gauss–Hermite Quadrature approximation (nAGQ = 25) and a random intercept for each subject. We conducted Bonferroni-adjusted pairwise post-hoc comparisons.

*Why did trustworthiness decrease when participants were interacting with a political opponent?* Bonferroni-adjusted pairwise comparisons (*F*(1.72, 591.76) = 69.04, *p* < 0.001, η_p_^2^ = 0.17) indicated that participants felt they *should* share the money to the same degree with a political affiliate as with a partner whose political affiliation was unknown (*M* = 5.65, *SE* = 0.11 versus *M* = 5.67, *SE* = 0.11, *p* = 1.00). However, when their interaction partner belonged to the “wrong” political camp, participants felt they *should* share the money significantly less (*M* = 4.65, *SE* = 0.13, both *p* < 0.001). More strikingly, those who kept the money and gave a political opponent nothing believed this was what they *should* do (see Fig. 2), the modal answer being 1 (“I should keep the $11”; 42.2%, *M* = 3.27, *SD* = 2.36). Thus, those who gave nothing back to Person A indicated that such untrustworthiness was the morally right thing to do towards a political opponent. In contrast, for those who shared the money with a political opponent, the modal answer was 7 (“I should keep $3.75 and send $3.75 back to Person A”; 67.1%, *M* = 6.22, *SD* = 1.41).

We calculated a new binary variable to measure *discriminatory trustworthiness* based on participants’ binary decisions to reciprocate trust to a same-party supporter versus an opposing-party supporter (i.e., only being trustworthy towards a political affiliate, but not towards a political opponent). Likewise, we built such difference scores for how participants felt they *should* behave as Person B. A binary logistic regression (adjusted for differences in what they wanted to do) showed that participants were more likely to display *discriminatory trustworthiness* the less they felt they *should* act trustworthily to the out-group compared to the in-group (*b* = 0.70, *OR* = 2.01, *p* < 0.001, 95% CI[1.49, 2.70]) (Here and in all subsequent regression analyses, estimates for continuous predictors are based on z-standardized variables).

*Summary.* Both trust and trustworthiness were seriously hampered by political partisanship. Thus, unlike in previous studies^20^, partisanship influenced not only trust but also trustworthiness, even though participants felt a strong obligation to be trustworthy towards an anonymous interaction partner^22,24^. What is more, those who did not reciprocate trust of a political opponent indicated that this behavior was ethically appropriate (see Fig. 2). All of these effects were shown for both Democrats and Republicans.

### Study 2

In Study 2, we let participants play our newly developed nastiness game (see Introduction), in which Person B has no material self-interest in not rewarding trustful behavior by Person A.

**Fig. 3:**
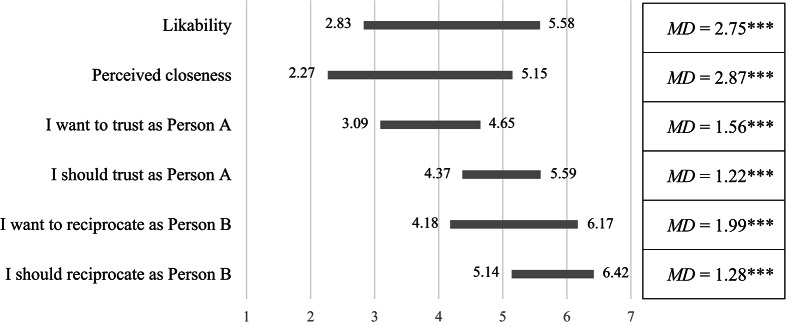
Rounded means (and mean differences) of participants’ perceptions of likability and closeness, as well as what participants wanted and felt they should do as Person A and B toward the political out-group and in-group. Higher values indicate greater likability, closeness, stronger *wanting* to trust (1 = *I want to keep the $2.50* to 7 = *I want to send the $2.50 to Person B*) and feeling one *should* trust (1 = *I should keep the $2.50* to 7 = *I should send the $2.50 to Person B*) as Person A and stronger *wanting* to reciprocate trust (1 = *I want to keep the $2.50 and send $0 to Person A* to 7 = *I want to keep $2.50 and send $3.75 back to Person A*) and feeling one *should* reciprocate trust (1 = *I should keep the $2.50 and send $0 to Person A* to 7 = *I should keep $2.50 and send $3.75 back to Person A*) as Person B. All values regarding the out-group are on the left side of the bar; all values regarding the in-group are on the right side of the bar. Asterisks indicate the significance of mean differences (in-group—out-group), as calculated using paired samples *t*-tests at the two-sided *p* < 0.05 level (*), at the *p* < 0.01 level (**), and at the *p* < 0.001 level (***).

As in Study 1, participants reported perceiving themselves as significantly closer to an interaction partner sharing their political affiliation than to one of an opposing party (*M* = 5.15, *SD* = 1.44 versus *M* = 2.27, *SD* = 1.31, *t*(493) = 36.62, *p* < 0.001, *d* = 1.65, 95% CI[1.54, 1.77]). Also, they indicated liking someone from the in-group significantly more than from the out-group (*M* = 5.58, *SD* = 1.15 versus *M* = 2.83, *SD* = 1.45, *t*(493) = 32.13, *p* < 0.001, *d* = 1.45, 95% CI[1.32, 1.58]). Thus, again participants indicated to not like their political opponents and they perceived themselves to be rather different (see Fig. 3).

**Fig. 4:**
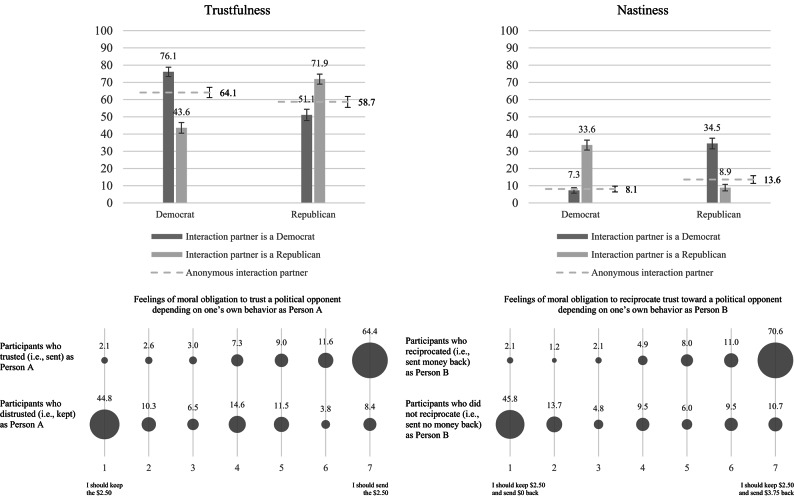
Proportion of participants deciding to send the money as Person A (upper, left) or for the interaction partner to receive no money as Person B (upper, right) by own party and the interaction partner’s party. The lower part of the Figure highlights feelings of moral obligation towards a political opponent as regards trust (lower, left) and reciprocity (lower, right). Error bars depict standard errors.

*Did participants trust their political opponents less than their political affiliates?* Indeed, overall, as Person A, more participants sent the money to a political affiliate (74.1%) than they sent the money to a political opponent (47.2%, χ^2^(1) = 123.57, *p* < 0.001) (see Fig. 4). Again, both Democrats and Republicans displayed out-group discrimination: Each trusted an interaction partner with an undisclosed affiliation significantly more than a political opponent (*OR* = 10.06, *p* < 0.001, 95% CI[4.36, 23.22] and *OR* = 2.36, *p* = 0.020, 95% CI[1.11, 5.03], respectively). Beyond that, Democrats and Republicans also trusted an anonymous interaction partner significantly less than a political affiliate, indicating in-group favoritism (*OR* = 0.21, *p* < 0.001, 95% CI[0.10, 0.49] and *OR* = 0.20, *p* < 0.001, 95% CI[0.09, 0.47], respectively).

Furthermore, participants expected a majority of 68.38% (*SD* = 24.72%) of all political affiliates to reciprocate their trust (i.e., not deprive them of getting $3.75 if they sent money to Person B). However, they only expected a minority of 36.15% (*SD* = 27.84%) of all political opponents to reciprocate their trust (*t*(493) = 21.70, *p* < 0.001, *d* = 0.98, 95% CI[0.86, 1.10]).

*Once more, participants were right in their skepticism towards political opponents.* When paired with a political affiliate, only a small minority of 8.1% acted in a nasty manner (i.e., they decided to give them no money). This value jumped to 34.0% when Person B was coupled with a political opponent (χ^2^(1) = 112.01, *p* < 0.001) (see Fig. 4). Both Democrats and Republicans decided significantly less often for someone of undisclosed political partisanship to receive no money at all than for a political opponent, thus displaying strong out-group discrimination (*OR* = 0.03, *p* < 0.001, 95% CI[0.01, 0.10] and *OR* = 0.09, *p* < 0.001, 95% CI[0.04, 0.23], respectively). To the contrary, neither Republicans nor Democrats displayed in-group favoritism. That is, they did not hand the money more often to a political affiliate than to an anonymous interaction partner (*OR* = 1.21, *p* = 1.000, 95% CI[0.42, 3.47] and *OR* = 2.34, *p* = 0.106, 95% CI [0.89, 6.15], respectively).

*Was participants’ perception of moral obligations influenced by the political preferences of their interaction partners?* Yes, it was. Bonferroni-adjusted pairwise comparisons (*F*(1.48, 729.95) = 130.01, *p* < 0.001, η_p_^2^ = 0.21) indicated that participants strongly felt that they *should* share the money with both a political affiliate as well as with a partner whose political affiliation was unknown (*M* = 6.42, *SE* = 0.06 versus *M* = 6.31, *SE* = 0.07, *p* = 0.143). However, when interacting with a political opponent, participants felt they *should* share the money significantly less (*M* = 5.14, *SE* = 0.11, both *p* < 0.001). Once again, behavior towards political opponents was strongly related to what Person B believed was the morally right course of action (see Fig. 4). Those who decided for Person A to get $3.75 felt obliged to do so with a modal value of 7 (“I should send $3.75 to Person A”; 70.6%) on the seven-point scale (*M* = 6.31, *SD* = 1.35). On the other hand, those who decided for Person A to get nothing felt they *should* not give any money to Person A, with a modal value of 1 (i.e., “I should send $0 to Person A”; 45.8%, *M* = 2.88, *SD* = 2.21).

As before, we calculated a binary variable to measure *discriminatory nastiness* (i.e., being nasty only toward a political opponent but not toward a political affiliate), and we likewise built difference scores for how participants felt they *should* decide toward Person A if they trusted them. A binary logistic regression (adjusted for differences in what they wanted to do) showed that participants were significantly more likely to display *discriminatory nastiness* the less they felt they *should* act so toward the out-group compared to the in-group (*b* = 0.98, *OR* = 2.66, *p* < 0.001, 95% CI[1.98, 3.58]).

*Summary.* We found strong evidence for both discriminatory trustfulness and discriminatory nastiness in our newly developed nastiness game. As Person A, only a minority of all participants trusted a political opponent, and about a third of all Persons B decided that Person A should go home empty-handed if they were coupled with a political opponent. Once again, it should be noted that Person B did not gain any material reward from such a decision. Instead, their behavior was driven by sheer nastiness (i.e., willingness to harm Person A). What is more, those who did not reciprocate a political opponent’s trust indicated that this behavior was ethically appropriate. Thus, for a substantial minority of all participants, to act in a hostile and antisocial manner appeared to be a moral act. Once again, we found no substantial differences between Democrats and Republicans.

### Study 3

Recently, a large-scale study has been conducted that aimed to test whether simple, short-term interventions can decrease polarization in the United States^39^. One intervention that stood out as quite successful was a commercial initially developed by a large brewery (although it never becomes apparent that the video is an advert for beer). In this commercial of four minutes and 24 seconds, one first hears six people separately state their political opinions on issues like transgenderism, climate change, and feminism. Then, they have to jointly assemble some furniture in pairs (actually, a counter to have a beer together). Once they finish these tasks, they are shown videos of the other person’s political statement (pairs are constructed so that they heavily disagree on one political issue). They are then given the choice to either leave or to discuss their political differences over a beer. All three pairs decide to stay and in a very appreciative manner they try to understand each other’s position^39^. In an earlier study^39^, showing this video to participants reduced partisan animosity considerably (*d* =−0.53). However, the intervention did not succeed in changing antidemocratic attitudes substantially.

In Study 3, we tested whether in the nastiness game, trust and reciprocity towards political opponents could be increased by such an intervention. To do so, we replicated and extended the paradigm of Study 2 by showing half of all participants the video described above, while the other half served as a control condition (i.e., did not see the video).

**Fig. 5:**
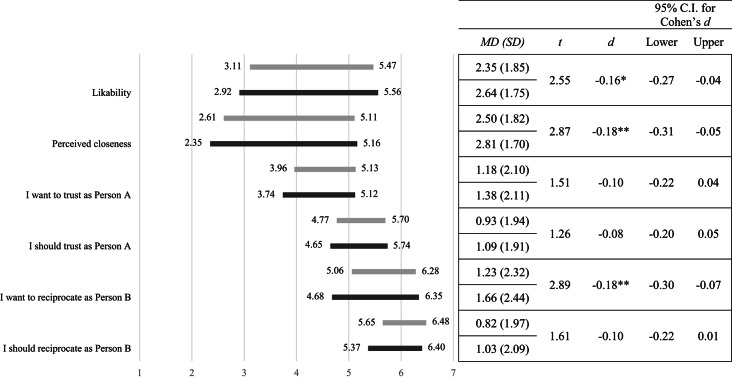
Mean differences of participants’ perceptions of likability and closeness, as well as what participants wanted and felt they should do as Person A and B toward the political out-group versus in-group by experimental condition. Higher values indicate greater likability, closeness, stronger *wanting* to trust (1 = *I want to keep the $2.50* to 7 = *I want to send the $2.50 to Person B*) and feeling one *should* trust (1 = *I should keep the $2.50* to 7 = *I should send the $2.50 to Person B*) as Person A and stronger *wanting* to reciprocate trust (1 = *I want to keep the $2.50 and send $0 to Person A* to 7 = *I want to keep $2.50 and send $3.75 back to Person A*) and feeling one *should* reciprocate trust (1 = *I should keep the $2.50 and send $0 to Person A* to 7 = *I should keep $2.50 and send $3.75 back to Person A*) as Person B by *Positive Contact Video* condition (light grey) and control condition (dark grey). All values regarding the out-group are on the left side of the bar; all values regarding the in-group are on the right side. Asterisks indicate the significance of the difference in mean differences (in-group—out-group) by experimental condition, as calculated using independent samples *t*-tests at the two-sided *p* < 0.05 level (*), at the *p* < 0.01 level (**), and at the *p* < 0.001 level (***).

*Did the intervention influence the perceived likability and closeness of a political opponent?* Indeed, in accordance with previous research, participants reported perceiving themselves as significantly closer to the out-group in the video condition than participants in the control condition (*M* = 2.61, *SD* = 1.40 versus *M* = 2.35, *SD* = 1.29, *t*(990.99) = 3.10, *p* = 0.002, *d* = 0.20, 95% CI[0.07, 0.33]). Likewise, participants reported liking the out-group significantly more in the video condition than participants in the control condition (*M* = 3.11, *SD* = 1.34 versus *M* = 2.92, *SD* = 1.41, *t*(1000) = 2.27, *p* = 0.023, *d* = 0.14, 95% CI[0.02, 0.27]). That is, the experimental intervention worked as intended on affective measures of partisan perception (see Fig. 5). However, on our measures, the observed effects were rather small and notably smaller than those previously reported.

**Fig. 6:**
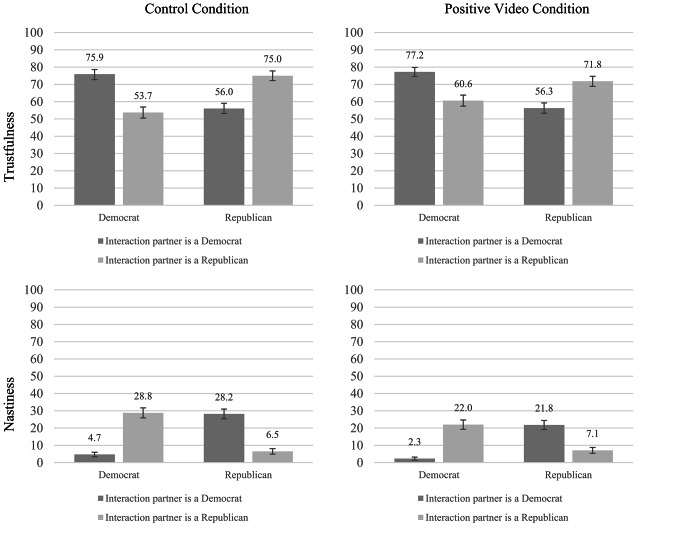
Proportion of participants deciding to send the money as Person A (upper) or for the interaction partner to receive no money as Person B (lower) by own party and the interaction partner’s party in the control (left) versus Positive Video condition (right). Error bars depict standard errors.

*Did the intervention also influence participants’ behaviors?* As regards trustfulness, no, it did not. In the control condition, 54.9% of all participants trusted a political opponent, 58.6% did so in the positive video condition—only a slight difference that was not significant (χ^2^(1) = 1.40, *p* = 0.237, φ = 0.04). However, the intervention influenced nastiness to some degree: In the control condition, 28.5% of all participants were nasty to a political opponent (i.e., as Person B, they deprived Person A of getting any money at all), compared to only 21.9% in the positive video condition (χ^2^(1) = 5.75, *p* = 0.017, φ = 0.08) (see Fig. 6).

However, when considering specifically *discriminatory nastiness (i.e., only being nasty to the political opponent but not their own),* there was no significant difference observed between the experimental conditions (*b* = -0.28, *OR* = 0.76, *p* = 0.075, 95% CI [0.56, 1.03]). In a similar way, there was no significant influence as to the difference whether participants felt they *should* reciprocate the trust of a co-partisan versus a counter-partisan (*M* = 1.03, *SD* = 2.09 versus *M* = 0.82, *SD* = 1.97, *t*(1000) = 1.61, *p* = 0.108, *d* = 0.10, 95% CI[−0.01, 0.22]).

*Summary*. In Study 3, we replicated previous studies’^39^ findings that the intervention increased political opponents’ likability and perceived closeness. However, these effects were smaller than in the earlier studies. More importantly, our intervention did not increase trustfulness and only moderately decreased the nastiness of our participants when they were paired with a political opponent.

## Discussion

Across three different studies, the willingness to trust another person in experimental games was substantially lower if that other person was either a political opponent, as compared to either a political co-partisan or a totally anonymous interaction partner (with no information about the other person’s political orientation). In all three studies, more than half of the participants did not trust a political opponent.

Likewise, the willingness to reciprocate another person’s trust was lower when that other person was a political opponent. This was the case if such untrustworthiness was in Person B’s own material self-interest (in the trust game in Study 1), but it was also true if Person B did not gain from letting Person A go home empty-handed (in the nastiness game in Studies 2 and 3).

Across all studies and all measured behaviors, we found strong and consistent evidence of out-group discrimination (in 8 out of 8 cases), but only inconsistent evidence of in-group favoritism (in 3 out of 8 cases). Thus, our participants did not treat interaction partners of their own political camp systematically better, but they did treat political opponents systematically worse. Findings from both cross-cultural research and laboratory experiments have shown that ingroup favoritism might be driven simply by the willingness to support people similar to oneself. Contrary, outgroup discrimination is indicative of a hostile attitude often based on perceived conflict and feeling threatened by the other side^40–42^.

Evidently, our research is not without its limitations. One might argue that experimental games are abstract and artificial^43–45^. However, the decisions of our participants were not hypothetical, but (for every tenth participant) for actual money. It might be criticized that we paid only every tenth participant actual money and that the stake sizes were relatively small. Each could be varied systematically in future studies. Although research indicates that neither paying all participants^35–37^ nor increasing stakes fundamentally changes participants’ behavior in experimental games^46–49^.

Unlike in most previous studies, we deliberately used a within-subjects design to measure whether participants actively discriminated against their political opponents. This might have made the norm to treat political co-partisans and political opponents alike rather salient. Yet, those participants who discriminated nevertheless did not do so with a bad conscience, but they were convinced that not being trustworthy towards a political opponent was the right (i.e., the most ethical) thing to do. In future studies, it might be worthwhile to systematically investigate whether results are independent of using such a within-subjects design and a between-subjects design, respectively.

Another limitation of the current study lies in the fact that it was only conducted in the United States. It is an open question to what degree our results can be generalized to other countries that might be less polarized than the United States and have more than two political parties in the national parliament.

Still, we would argue that the paradigms used in the present research capture important and general elements of social life: Am I willing to reciprocate trust given to me by another person and do I trust the other person to do the same with me?

Notably, across all studies, we found very few differences between Democrats and Republicans in terms of their trustfulness or trustworthiness (Study 1) or nastiness (Studies 2 and 3). Although consistent with previous research^50^, Democrats disliked Republicans even more than Republicans disliked Democrats in all three studies (see OSF).

If one were to extrapolate our results to real life, they would imply that in a majority of all cases, people shy away from having to trust others if they have different political preferences to their own. Moreover, in about a third of all interactions, even if they are trusted by someone on the other side of the political fence, Americans do not follow rules of kindness and courtesy if they deal with a person who they know to be voting for the “wrong” party.

Remarkably, such moralistic aggressions were not committed with a bad conscience, but with the conviction that both not being trustworthy towards and not trusting a political opponent is the morally right thing to do. Thus, moral obligations to trust others and reciprocate their trust with one’s own trustworthiness did not apply to political opponents.

How is it that at least a substantial minority of both Republicans and Democrats not only felt entitled but even obliged to harm political opponents actively, even if they did not profit from such behavior? Research on the antecedents of intergroup conflict show that hostile intergroup behavior is governed by negative beliefs (e.g., “they are evil” or “their goal is to eliminate us”), negative emotions (e.g., “I dislike them”) and exclusionary convictions (e.g., “they don’t deserve a fair treatment, as they are not fair themselves”)^51^. Such a conflict-supporting mindset^51^ will be strengthened if members of one’s in-group support one another’s positions, thereby radicalizing one another over time. Prejudice will turn into behavior when political opponents not only dislike each other but also justify discriminatory (antisocial) behavior from their cognitive and affective convictions. Furthermore, one’s own antisocial behavior can be regarded as preemptive self-defense, while the same behavior of the other side is seen as pure aggression^52^. Consequently, political opponents are regarded as falling outside one’s moral circle^53–55^.

In line with this reasoning, participants in our studies who discriminated against their political opponents did not do so with a bad conscience, but with full conviction. They were convinced that they *should* do so. This effect was quite stable even when (in Study 3) participants saw a video that stimulated positive feelings towards their political opponents (albeit admittedly only weakly). Political discrimination cannot easily be altered for those who perceive such discrimination as righteous and necessary.

Unfortunately, such tendencies are likely to form a vicious cycle. If someone is not trusted by another person or their trust is not reciprocated, this will be regarded as an insult, which will lead to a decrease in one’s trustfulness and trustworthiness in the future, which might in turn be regarded as an insult by the next person.

Citizens of the USA pay a price for political polarization, a price arguably much higher than the amount of money our participants did not earn because they refused to cooperate with each other. Regarding someone who votes for the “wrong” candidate as not deserving respect, trust, nor trustworthiness, poisons the relationship between strangers and colleagues at the workplace, and does harm to friendships, families, and intimate relationships.

## Methods

We conducted three studies on how political affiliation affects trust and reciprocity. Study 1 tested decisions in a binary trust game. Studies 2 and 3 used a new *nastiness game*, which differs from the standard trust game in that Person B has no material gain from withholding reciprocity. Across all studies, each participant interacted with a real co-partisan, opponent, or a partner with unknown affiliation, allowing a clear test of discrimination (i.e., when they acted trustfully or reciprocated trust to a co-partisan, but not to an opponent). Study 3 also tested whether an intervention could reduce discrimination. Participants from previous studies were excluded from participating in the subsequent studies. Participants were compensated in British pounds (GBP) through the Prolific platform. To enhance clarity for an international audience, monetary values are reported in U.S. dollars (USD) throughout the manuscript; the original GBP amounts are provided in the initial description and all subsequent references use the converted USD values for better readability. All analyses reported (i.e., Chi-squared tests, paired and independent *t*-tests, and binary logistic regression analyses) used two-sided tests. We report Student’s or Welch’s *t*-tests where appropriate based on Levene’s test of equal variances. All studies and target sample sizes were pre-registered. Materials, data, code, and pre-registrations are available at: https://osf.io/p5y4c/overview?view_only=c3574d89c7924d63bb5fdd3ac3b585c4.

### Study 1

#### Sample

We recruited a total of 402 participants via the online crowdsourcing platform Prolific at the end of August 2024. . Eligible participants had to reside in the U.S., identify as either Democratic or Republican voters and indicate that English was their first language. We excluded 56 participants from the analysis due to incorrectly answered control questions. Our final analysis sample consisted of 346 participants (168 women, 175 men, and three who did not want to specify) aged between 18 and 77 years (*M* = 38.99, *SD* = 12.38). Of those, 180 (52%) participants identified themselves as Democrats and 166 (48%) identified as Republicans. All participants received a flat payment of $1.60 (£1.30). Additionally, every tenth participant received a bonus according to one of their decisions in the decision-making situation.

#### Procedure

This study employed a 2 (participants’ party affiliation: Democrat vs. Republican) × 3 (interaction partners’ party affiliation: no info vs. in-group vs. out-group) design. The participants’ party affiliation acted as a quasi-experimental between-subjects factor, while the information about the interaction partners’ party affiliation served as an experimental within-subjects factor. Thus, each participant interacted with three different partners.

We relied on a version of the trust game—involving two participants (Person A and B)—to measure trust and cooperation^22,56^ with respect to the three interaction partners. In our game, Person A received an initial endowment of $2.50 (£2.00) and had two options: 1) Retain the money, in which case Person B received nothing or 2) send the money to Person B. If Person A sent the money to Person B, Person B had two options: 1) Keep all the money—$11 (£9.00)—for themselves, with Person A receiving no money at all; or 2) opt to receive $3.75 (£3.00) and ensure Person A also received $3.75 (£3.00). For each interaction partner, participants first responded to questions regarding their role as Person B, followed by their responses in the role of Person A.

Every participant first engaged in the game entirely anonymously, with no information about the political identity of their counterparts, and answered three control questions regarding the potential monetary outcomes of the decision-making situation. Then, in the role of Person B, participants were asked to estimate the percentage of Person A participants who would decide to keep the $2.50 or send the $2.50 to Person B. Before participants made their conditional decision as Person B (either keep the $11 *or* keep $3.75 and send $3.75 back to Person A), they were asked how they *wanted* to decide in their role as Person B on a scale from 1 = *I want to keep the $11* to 7 = *I want to keep $3.75 and send $3.75 back to Person A,* and how they thought they *should* decide on a scale from 1 = *I should keep the $11* to 7 = *I should keep $3.75 and send $3.75 back to Person A* as a measure of moral obligation^24^.

Subsequently, in their role as Person A, participants were asked for the percentage of Person B participants who would decide to keep the $11 for themselves or keep $3.75 and send $3.75 back to Person A. This percentage was used to measure participants’ cognitive trust in others’ trustworthiness^57^. Again, before participants made their decision as Person A (either keep the $2.50 *or* send the $2.50 to Person B), they were asked to state how they *wanted* to decide on a scale from 1 = *I want to keep the $2.50* to 7 = *I want to send the $2.50 to Person B,* and how they thought they *should* decide on a scale from 1 = *I should keep the $2.50* to 7 = *I should send the $2.50 to Person B*.

Afterward, all participants were asked for their political identity and were truthfully informed that they would engage in the same situation twice more, once with a Democrat and once with a Republican. Thus, in random order, they interacted with a partner who shared their own party and one with a different party affiliation. Before engaging in the decision-making situations with these interaction partners—which included the same questions in the same order as those in the anonymous interaction but with an explicit reference to the interaction partner’s political affiliation—participants were asked to rate how much they believed they had in common with same-party and opposing-party supporters, respectively, using an adapted version of the Inclusion of Other in the Self Scale^58^ and how likable they perceived them using a scale consisting of six items presented in random order. For example, when the interaction partner was identified as a Democrat, participants answered to “I am fond of Democrats,” “I admire Democrats,” “I like Democrats,” “I feel angry towards Democrats,” “I feel irritated by Democrats,” “I feel annoyed by Democrats” on a scale from 1 = *strongly disagree* to 7 = *strongly agree* (*M*_*D*_ = 4.08, *SD*_*D*_ = 1.63, *α*_*D*_ = 0.94; *M*_*R*_ = 3.97, *SD*_*R*_ = 1.91, *α*_*R*_ = 0.96, with high values indicating higher likability after reverse coding of appropriate items). Perceived closeness and likability were assessed randomly before engaging in the decision-making situation with a partner from the respective party. Finally, participants were asked for their gender and age.

We operationalized discrimination as inconsistent behavior toward both interaction partners, specifically by acting positively (i.e., trusting, reciprocating trust) toward someone supporting one’s own party and negatively (i.e., not trusting, not reciprocating trust) toward the political opponent. In this study and subsequent ones, we did not find a significant main effect of gender on discriminatory reciprocity (see OSF).

### Study 2

#### Sample

We recruited a total of 601 participants via Prolific at the beginning of September 2024. Again, eligible participants had to reside in the U.S., identify as either Democratic or Republican voters, and indicate English as their first language. We excluded 107 participants from the analysis due to incorrectly answered control questions. Thus, our final analysis sample comprised 494 participants (245 women, 244 men, five did not want to specify) aged between 18 and 78 (*M* = 42.40, *SD* = 13.46), with 259 (52.4%) participants identifying as Democratic voters and 235 (47.6%) as Republican voters. All participants received a flat payment of $1.45 (£1.15). Again, every tenth participant received a bonus according to one of their decisions in the decision-making situation.

#### Procedure

Overall, this study replicated the design of Study 1. We employed a 2 (participants’ party affiliation: Democrat vs. Republican) × 3 (interaction partners’ party affiliation: no info vs. in-group vs. out-group) design. Once again, the participants’ party affiliation acted as a quasi-experimental between-subjects factor, while the information about the interaction partners’ party affiliation served as an experimental within-subjects factor.

In this study, we asked the same questions as in Study 1. However, we now used a variation of the trust game where Person B had no apparent monetary incentive not to reciprocate trust — the *nastiness game*. In this variation, Person A received an initial endowment of $2.50 (£2.00) and had two options as before: 1) Keep the money, in which case Person B received no money; or 2) give the money to Person B. If Person A sent the money to Person B, Person B received the $2.50 (£2.00) regardless of any further decisions. In that case, Person B decided at no personal cost whether Person A received 1) nothing ($0); or 2) an amount of $3.75 (£3.00) which is higher than their initial sum.

Questions regarding participants’ estimations of how trustful Persons A were in general, what they *want* to do and thought they *should* do as Person B, their decisions as Person B, their estimations of Persons’ B reciprocity, what they *wanted* to do and thought they *should* do as Person A, and their decisions as Person A were all adjusted to reflect the new monetary outcomes. Apart from these changes, we followed the same procedure as in Study 1.

### Study 3

#### Sample

We recruited a total of 1198 participants via Prolific in mid-September 2024. Again, eligible participants had to reside in the U.S., identify as either Democratic or Republican voters, and indicate that English was their first language. We excluded 196 participants from the analysis due to incorrectly answered control questions. Our final analysis sample thus consisted of 1002 participants (495 women, 495 men, 12 did not want to specify) aged between 18 and 82 years (*M* = 43.10, *SD* = 14.08). Of those, 516 (51.5%) participants identified themselves as Democrats, and 486 (48.5%) as Republicans. All participants received a flat payment of $2.00 (£1.60). Additionally, every tenth participant received a bonus according to one of their decisions in the decision-making situation.

#### Procedure

This study essentially replicated the design of Study 2. However, in addition, participants were now experimentally assigned to either view a *Positive Contact Video* (*n* = 497) or nothing (*n* = 505), after engaging in the anonymous decision-making situation (and having indicated their political identity), but before responding to the questions and making decisions involving a Democratic and a Republican voter. The video depicted three pairs of people working on a common task and connecting despite different political orientations (see OSF). A recent study identified this adapted intervention as very effective in reducing partisan discrimination^39^. Participants had to remain on the page showing the video until the estimated watching time had passed and answer three multiple-choice questions about the content afterward to check for attentive watching. If assigned to this condition, participants were truthfully told that their interaction partner watched the same video. Apart from the video presentation, we followed the same procedure and measured the same variables as in Study 2.

## Data Availability

The pre-registrations, materials, data, and code for the studies and results reported in this paper are available at: https://osf.io/p5y4c/overview?view_only=c3574d89c7924d63bb5fdd3ac3b585c4.
